# Comparison of seven models for the progression patterns of multiple chronic conditions in longitudinal studies

**DOI:** 10.1136/bmjph-2024-000963

**Published:** 2024-11-29

**Authors:** Mohammad Reza Baneshi, Gita Mishra, Annette Dobson

**Affiliations:** 1 School of Public Health, The University of Queensland Faculty of Medicine, Herston, Queensland, Australia

**Keywords:** Epidemiology, methods, Public Health, Comorbidity, Statistics as Topic

## Abstract

**Introduction:**

Studies investigating the relationship between patterns of multimorbidity and risk of a new condition have typically defined the patterns at a baseline time and used Kaplan-Meier (KM) or Cox proportional hazards regression. These methods do not consider the competing risk of death or the changes in the patterns of conditions over time. This study illustrates how these methodological limitations can be overcome in the setting of progression from cardiometabolic conditions to dementia.

**Methods:**

Data from 11 930 women who participated in the Australian Longitudinal Study on Women’s Health were used to define patterns of diabetes, heart disease and stroke and estimate the cumulative incidence or HRs of subsequent dementia. Seven methods were compared. For cumulative incidence these were KM method, cumulative incidence function (CIF) (to account for the competing risk of death) and multistate model with Aalen-Johansen estimates (to account also for the progression of conditions over time). For HRs, the corresponding methods were Cox model and Fine and Gray model (for sub-HRs) with the cardiometabolic patterns treated as time-invariant (from baseline) or as time-varying predictors.

**Results:**

The estimated cumulative incidence of dementia using the KM method declined when the competing risk of death was considered. For example, for women with no cardiometabolic condition at baseline, the KM and CIF estimates were 35.7% (95% CI 34.6%, 36.8%) and 27.3% (26.4%, 28.2%) but these women may have developed cardiometabolic conditions during the study which would increase their risk. The Aalen-Johansen multistate estimate for women with no cardiometabolic condition over the whole study period was 11.0% (10.4%, 11.7%). Comparing models to estimate HRs, the estimates in the Fine and Gray models were lower than those in the Cox models.

**Conclusions:**

Multistate and time-varying survival analysis models should be used to study the natural development of multimorbidity.

**WHAT IS ALREADY KNOWN ON THIS TOPIC**

- Previous studies have defined the patterns of multimorbidity based on reports of multiple chronic conditions at a baseline time and employed standard survival analysis methods to estimate the risk of diagnosis of a new condition. Specifically, they have used the Kaplan-Meier (KM) method to estimate the cumulative incidence or the Cox proportional hazards model to estimate HRs. These methods do not consider the competing risk of death or changing risk if the patterns of multimorbidity change after the baseline.

**WHAT THIS STUDY ADDS**

- These standard methods overestimate cumulative incidence and HRs. The effects were illustrated using data from a 25-year population-based longitudinal study showing the progression of cardiometabolic multimorbidity (from diabetes, heart disease and stroke) and the risk of dementia. For cumulative incidence, multistate models with Aalen-Johansen estimates can be used to account for competing risks (such as death) and changes in risk that may occur with changing patterns of multimorbidity. For HRs competing risk models, such as that proposed by Fine and Gray, with time-varying predictors can be used to take into account the progression of multimorbidity.

**HOW THIS STUDY MIGHT AFFECT RESEARCH, PRACTICE OR POLICY**

- Estimates of cumulative incidence and HRs of disease progression in the context of multimorbidity must take into account both the competing risk of death and changes in patterns of disease that can affect the risk of further chronic disease.

## Introduction

 The association between multimorbidity, defined as the existence of at least two chronic conditions, and poor health outcomes and greater use of health services has been well-documented.^1^ Multimorbidity and increasing age may put people at higher risk of additional chronic conditions.^2^ For example, several studies have investigated the relationship between patterns of multimorbidity (eg, patterns of cardiometabolic multimorbidity) and the risk of being diagnosed with a new condition (eg, dementia).^3^,^7^ But as Hu has recently pointed out the association will be affected by both the competing risk of death and the changes in the patterns of multimorbidity over time or as the study population ages.^8^

The commonly used analytic approach involves defining the patterns of multimorbidity at a baseline time and estimating the risk of diagnosis with a new condition using standard survival analyses such as the Kaplan-Meier method (KM) (to estimate cumulative incidence) or the Cox proportional hazards regression model (to estimate HRs). These methods have drawbacks. The first limitation is the failure to consider the competing risk of death. A competing risk is an event whose occurrence precludes the occurrence of the primary outcome of interest.^9^ For example, death is a competing risk that changes the probability of diagnosis with other conditions.^10^ The second limitation is that the progression of conditions between the baseline and the occurrence of the outcome is not captured.^8^ Often, the incidence of conditions increases with age. Therefore, if the subjects are recruited at different ages, the distribution of the conditions and hence the patterns of multimorbidity will differ.^11^

This paper describes the statistical methods that can be used to overcome these limitations and shows how they reduce bias when the aim is to estimate the cumulative incidence or improve the goodness of fit when the aim is to estimate HRs associated with different patterns of multimorbidity.

For illustration purposes, this research examines the risk of incident dementia (as the outcome) for subjects with different progression patterns of cardiometabolic multimorbidity (as the exposures). For simplicity, covariates (such as smoking) are not considered. Dementia was selected as the main outcome as this disease is a potential accelerator of death.^12^ Cardiometabolic conditions were selected as exposure variables as the association between these conditions and the risk of dementia has been confirmed in several studies.^13 14^

As a case study patterns of multimorbidity are illustrated by combinations of three cardiometabolic conditions diabetes (D), heart disease (H) and stroke (S). At any time, the study participants can be categorised into eight mutually exclusive patterns: no disease (none), diabetes only (D), heart disease only (H), stroke only (S), diabetes and heart disease (D+H), diabetes and stroke (D+S), heart disease and stroke (H+S), and diabetes and heart disease and stroke (D+H+S). Seven different methods of analysis are explained and illustrated using data from participants in the Australian Longitudinal Study on Women’s Health (ALSWH).

## Materials and methods

Survival analysis is based on the time (shown by t) from a baseline until the occurrence of an event of interest. Two main functions in survival analysis are the hazard function (shown by h) and the survival function (shown by S). In the absence of competing risks, the hazard function describes the instantaneous rate of occurrence of the event of interest in subjects who are still at risk of the event. The survival function at time t describes the probability of being event-free at least up to time t. Technical details to estimate hazard and survival functions (ie, h and S) are provided in online supplemental materials.

### Estimation of the cumulative incidence

The KM method gives a simple estimate of the survival function. The KM method relies on the assumption of non-informative censoring. This means that individuals who are censored (ie, are no longer contributing data after time t) are assumed to have the same future risk of the outcome. This assumption is violated when the reason for censoring is the occurrence of a competing event such as death. Therefore, KM estimates tend to be biased upward.^15 16^

More generally, in the presence of competing risks, a person can experience one of K different events (including the outcome of interest). The cumulative incidence function (CIF) method allows for the estimation of the cumulative incidence while taking the competing risks into account. Assume status is an indicator variable denoting the type of event that occurred. CIF for the kth event (eg, dementia) is an estimate of the probability of experiencing the kth event before time t and before the occurrence of any competing events (eg, death) (see online supplemental materials for technical details).

In contrast to the competing risk models in which people can only experience one of the outcomes of interest or a competing event, the multistate model provides a flexible framework that captures the progression of events and allows transition between several events over the life course.^17^ The term state is used to describe any one or combination of events or conditions (eg, corresponding to different multimorbidity patterns). Multistate models are described by two main quantities: transition hazard and transition probability. The hazard for the transition between one state and the next (say from state m to state n at time t) is the instantaneous rate of occurrence of the second state among people who are in the first state.^18^ The estimated transition hazards are then used to obtain the Aalen-Johansen estimates of the cumulative incidence.^17^ This differs from the KM and CIF methods which estimate the cumulative incidence of the outcome from baseline pattern (ie, ignoring changes in these patterns that may have occurred during the study period).

### Estimation of the HR

The Cox proportional hazards regression model relates the hazard function (ie, rate of occurrence of the outcome) to the independent variables (eg, baseline patterns of multimorbidity). Like the KM method, the Cox regression relies on the assumption of noninformative censoring. To overcome this limitation, competing risk models can be used such as that suggested by Fine and Gray.^9^ They defined a subdistributional hazard function to describe the instantaneous rate of occurrence of the kth event in people who have not yet experienced this event including those who have experienced a competing event. The estimated HRs should be interpreted in terms of the relative incidence of the outcome rather than hazards.

Both Cox and Fine and Gray models can be used to estimate relative hazards of the outcome of people with various combinations of conditions at baseline compared with the rate for people with none of these conditions. However, these analyses ignore the progression of multimorbidity during the study period. This limitation can be overcome using the time-varying Cox regression and time-varying Fine and Gray models. For these models, the patterns of conditions are treated as time-varying covariates.^19^

### Study sample

ALSWH is a prospective nationwide population-based study of four cohorts born in 1989–1995, 1973–1978, 1946–1951 and 1921–1926.^20^ The current analyses related to women born in 1921–1926 who consented for data linkage.^21^ The baseline survey was conducted in 1996, when the women were aged 70–75 years and the next surveys were conducted in a 3-year cycle up to 2011. Since November 2011, the women have been surveyed every 6 months. Data on the first record of cardiometabolic conditions (ie, diabetes, heart disease and stroke) and dementia have been obtained from multiple sources, including hospital and medication records, aged care assessments, death certificates and survey data completed by the participants or other informants (online supplemental table S1). Diabetes included type 1 and 2 diabetes mellitus and excluded gestational diabetes. Heart disease included heart surgery (heart bypass, angioplasty and angiography) and acute coronary syndrome but not heart failure. Stroke included ischaemic and haemorrhagic stroke. Dementia included Alzheimer’s dementia, vascular dementia and unspecified dementia. For data analysis, it was assumed when a condition was reported, it continued on for the whole follow-up period. This assumption may be realistic due to the chronic nature of the conditions.

Of the 12 432 women who participated in the first ALSWH survey in 1996, 12 070 women consented to data linkage. Removing 125 women for whom the first record of a condition was after or the same as the date of death and another 15 women with a record of dementia before the baseline survey, the sample size for data analysis was 11 930. The final date for follow-up was 31 December 2019 (when the surviving women would have been aged 93–98).

### Statistical analysis

Seven methods of analysis were used: three methods to estimate the cumulative incidence of dementia (and competing events) up to the age of 90, and four methods to estimate HRs. Methods applied, the measurement of the exposure variables (ie, patterns of cardiometabolic multimorbidity), and the limitations of each method are summarised in table 1. The different methods need different data layouts and time metrics. An example of the appropriate data layout for different methods is provided in online supplemental materials.

**Table 1:** List of methods applied, measurement of exposure and limitations

Measure of risk	Model	Outcome: time to dementia		Exposure	Limitation
		Time	Status	Patterns of cardiometabolic multimorbidity	
Cumulative incidence	Kaplan-Meier	Age at dementia for those with a record of dementiaAge at death for women who died without report of dementiaAge on 31 December 2019 for other women	1 for women with a record of dementia0 otherwise	A	CD
	Cumulative incidence function (CIF)	Age at dementia for those with a record of dementiaAge at death for women who died without report of dementiaAge on 31 December 2019 for other women	1 for women with a record of dementia2 for women who died without a record of dementia0 otherwise	A	D
	Aalen-Johansen (multistate model)	The time to reach each state was set as the age in that state	1 for women who moved to each state0 otherwise	B	Neither
HR	Cox model	Difference between the last follow-up time (ie, age of dementia, death or censoring whichever was reported first) and the age at baseline	1 for women with a record of dementia0 otherwise	A	CD
	Fine and Gray model (for sub-HRs)	Difference between the last follow-up time (ie, age of dementia, death or censoring whichever was reported first) and the age at baseline	1 for women with a record of dementia2 for women who died without a record of dementia0 otherwise	A	D
	Extended Cox model with time-varying covariates	the start and stop variables were used to define the periods at which women contributed to the analysis with different progression patterns	For the last start and stop period:(1 for women with a record of dementia0 otherwise)For other start and stop periods: (0)	B	C
	Fine and Gray model (for sub-HRs) for time-varying covariates	The start and stop variables were used to define the periods at which women contributed to the analysis with different progression patterns	For the last start and stop period:(1 for women with a record of dementia2 for women who died without a record of dementia0 otherwise)For other start and stop periods: (0)	B	Neither

A: the status of diabetes, heart disease and stroke was determined at the baseline survey. Women were classified into eight mutually exclusive categories.

B: the progression of diabetes, heart disease and stroke over time was considered. Therefore, there was no need to determine the baseline pattern.

C: the competing risk of death was not considered.

D: the progression of cardiometabolic conditions during the study period was not considered.

The KM method was used to estimate the cumulative incidence of dementia from the baseline combinations of D, H and S. In the KM method, status was defined as 1 for women with a record of dementia and 0 otherwise. Time was defined as the age at dementia for those with a record of dementia, or the age at death or censoring (ie, age on 31 December 2019) for other women.

The CIF method was used to estimate the cumulative incidence of dementia from the baseline combinations of D, H and S while considering the competing risk of death. In the CIF method, status was defined as 1 for women with a record of dementia, 2 for women who died without a record of dementia and 0 otherwise. Time was defined as the age at the corresponding events.

Neither KM nor CIF methods capture the progression of conditions (and hence the progression of multimorbidity patterns). The multistage model with the Aalen-Johansen method was used to estimate the transition probabilities between all states (more details in online supplemental materials), and the cumulative incidence of dementia (and all other competing events) from the last combination of D, H and S. State was defined as a condition or combination of conditions (eg, ‘diabetes’ or ‘diabetes and stroke’). Death was defined as the absorbing state. Cox regression with separate baseline hazard functions for each transition was used to estimate the transition hazards for all possible transitions (more details in online supplemental materials). To capture the effect of ageing and holding the Markov assumption, the survival time is calculated from the beginning of the study (called clock-forward) rather than the age at entry to the states (called clock reset). In other words, the time to reach each state was set as age in that state.

Standard Cox and Fine and Gray methods were used to estimate HRs of the baseline combinations of D, H and S compared with no baseline conditions, ignoring changes in the patterns during the study period. For these analyses, time was defined as the difference between the last follow-up time (ie, age of dementia, death or censoring whichever was reported first) and the age at baseline. In the standard Cox method, status was defined as 1 for women with a record of dementia and 0 otherwise. In the Fine and Gray model, status was defined as 1 for women with a record of dementia, 2 for women who died without a record of dementia and 0 otherwise.

Finally, time-varying Cox and Fine and Gray methods were applied to take the changing patterns into account, with the multimorbidity pattern treated as a time-varying covariate. In time-dependent models, start and stop variables were used to define the periods at which women contributed to the analysis with different progression patterns. For example, for a woman with a report of diabetes at the age of 75, stroke at the age of 77 and dementia at the age of 85, the following intervals have been defined: (0, 75), (75, 77) and (77, 85) (more details in online supplemental materials). The status was defined for each follow-up window. In the time-dependent Cox model, for the last start and stop window, status was coded as 1 for women with a record of dementia and 0 otherwise. Status took 0 for other start and stop intervals. In the time-dependent Fine and Gray model, for the last start and start follow-up interval, status was coded as 1 for women with a record of dementia, 2 for women who died without a record of dementia and 0 otherwise. For all four regression models, the Bayesian information criterion (BIC) was estimated as a measure of the goodness of fit. Status took 0 for other start and stop windows.

### Assumptions

The proportional hazard assumption was checked using the test of interaction with time. Moreover, an assumption behind multistate and time-dependent models is that no more than one event per person can happen at the same time. In our data, some patients had two conditions reported on the same date (ranging from 59 (0.5%) for ischaemic heart disease (IHD) and dementia to 184 (1.5%) for IHD and stroke) (known as tie). When a tie occurred, ties were split randomly. As a sensitivity analysis, the analysis was repeated excluding the ties and there were only minor changes to decimal points in the estimated risks.

### Software

The following packages in R were used for data analysis: ‘tidyverse’^22^ and ‘lubridate’^23^ for data cleaning and preparation, ‘ggplot2’ for visualisation,^24^ ‘survival’ for KM, standard Cox and time-varying Cox models, ‘tidycmprsk’ for estimation of CIF,^25^ and ‘mstate’ for the multistate models.^18^

## Results

### Patterns of cardiovascular multimorbidity

Of the 11 930 women in the study sample, 83.8% had no cardiometabolic condition at baseline (ie, at ages 70–75) and the proportions with patterns of ‘S’, ‘D+S’ and ‘D+H+S’ were all below 1.0% (table 2). To show the effects of age on the changing patterns of conditions, corresponding numbers based on the reports of conditions up to the ages 76–81 (ie, age at survey 3) and ages 82–87 (ie, age at survey 5) are given in online supplemental tables S2 and S3. For example, the proportion of women with the pattern of ‘S’ was 101 (0.8%) at the baseline survey (ages 70–75 years), 419 (3.6%) at survey 3 (ages 76–81 years) and 546 (5.3%) at survey 5 (ages 82–87 years).

**Table 2:** Frequency (percentages) of women with each pattern of cardiometabolic condition at baseline (age 70–75)

	Women (n)	New cases of dementia (n)	Deaths (n)
None	10 000 (83.8%)	3662 (36.6%)	8187 (81.9%)
D	699 (5.9%)	255 (36.5%)	641 (91.7%)
H	798 (6.7%)	292 (36.6%)	726 (91.0%)
S	101 (0.8%)	38 (37.6%)	92 (91.1%)
D+H	164 (1.4%)	40 (24.4%)	157 (95.7%)
D+S	22 (0.2%)	8 (36.4%)	21 (95.5%)
H+S	119 (1.0%)	45 (37.8%)	110 (92.4%)
D+H+S	27 (0.2%)	14 (51.9%)	26 (96.3%)
Total	11 930	4354	9960

D, diabetes; H, heart disease; S, stroke

Online supplemental figure S1 depicts the progression of cardiometabolic conditions over the study period and before the first report of dementia or death. A total of 3786 women (31.7%) did not have a report of any cardiometabolic condition by the end of the study. The number (proportion) of women with each cardiometabolic condition as the first condition were ‘D’ 1884 (15.8%), ‘H’ 4840 (40.5%) and ‘S’ 1420 (11.9%). Among states of two conditions, the most common was 1476 women (12.4%) whose first and second reported conditions were ‘D’ and ‘H’ in either order (ie, ‘D+H’). Moreover, 534 women (4.5%) had all three conditions before having a report of dementia or death (state of ‘D+H+S’ in online supplemental figure S1). These figures (that contributed to the multistate model) were much higher than the figures where the pattern was defined based on the history of conditions up to a particular survey.

### Estimates of cumulative incidence

**Figure 1:**
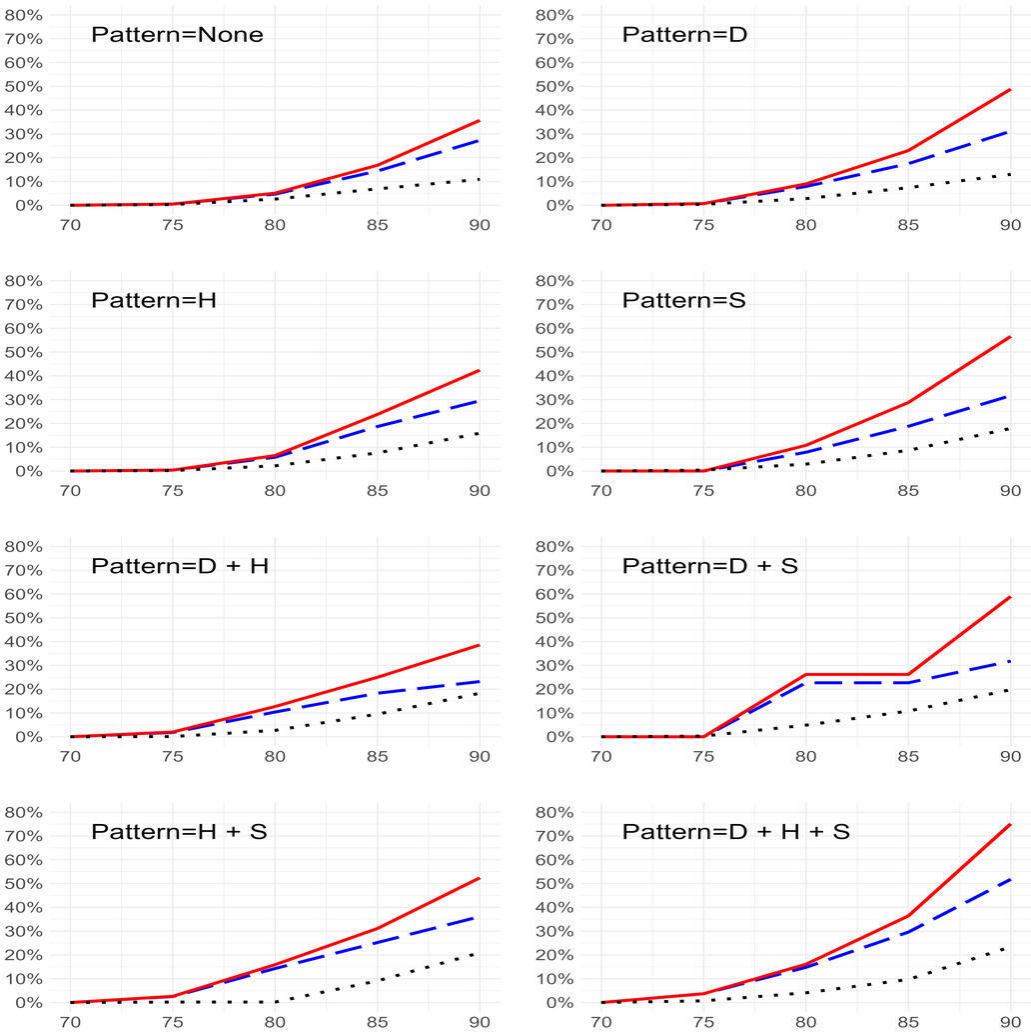
Comparison of estimated cumulative incidence of dementia for women with different patterns of cardiometabolic multimorbidity using different methods; Kaplan-Meier (red solid line), cumulative incidence function (blue dashed line) and multistate model (black dotted line).

The estimated cumulative incidence of dementia at different ages for all three methods and all eight patterns is depicted in figure 1. Estimates from the KM and CIF were similar up to the age of 80. After the age of 80, the KM estimates increased at a faster rate. Estimates from the multistate model were lower than those from the other two methods.

The estimated cumulative incidence of different outcomes using different methods is summarised in table 3. For example, for women with the pattern of ‘None’, the only possible outcomes in the KM and CIF methods were dementia or death (since these methods defined the pattern based on baseline reports and did not consider the progression of cardiometabolic conditions). However, in the multistate model, the possible outcomes from the pattern of ‘None’ were ‘D’, ‘H’, ‘S’, ‘dementia’ or ‘death’.

**Table 3:** Cumulative incidence of cardiometabolic conditions, dementia and death up to the age of 90 for women with different progression patterns by different methods

Method	Outcome	Kaplan-Meier method	Cumulative incidence function method	Aalen-Johansen method
None	D	a	a	15.6% (15.0%, 16.3%)
	H	a	a	38.5% (37.6%, 39.3%)
	S	a	a	10.7% (10.2%, 11.3%)
	Dementia	35.7% (34.6%, 36.8%)	27.3% (26.4%, 28.2%)	11.0% (10.4%, 11.7%)
	Death	50.6% (49.6%, 51.6%)	34.3% (33.4%, 35.3%)	10.5% (9.9%, 11.0%)
D	H	a	a	44.7% (42.5%, 47.0%)
	S	a	a	11.0% (9.7%, 12.6%)
	Dementia	48.8% (43.6%, 53.5%)	31.1% (27.8%, 34.6%)	13.0% (11.5%, 14.6%)
	Death	69.7% (66.1%, 72.9%)	47.6% (43.8%, 51.2%)	12.7% (11.3%, 14.3%)
H	D	a	a	11.4% (10.6%, 12.4%)
	S	a	a	16.2% (15.2%, 17.3%)
	Dementia	42.4% (38.0%, 46.5%)	29.5% (26.4%, 32.8%)	15.9% (14.9%, 16.9%)
	Death	65.5% (62.0%, 68.6%)	44.1% (40.6%, 47.5%)	25.5% (24.3%, 26.7%)
S	D	a	a	5.6% (4.4%, 6.8%)
	H	a	a	25.7% (23.5%, 28.1%)
	Dementia	56.6% (40.5%, 68.4%)	31.7% (22.7%, 40.9%)	17.9% (16.0%, 19.9%)
	Death	76.3% (66.3%, 83.3%)	50.4% (40.2%, 59.8%)	25.4% (23.2%, 27.8%)
D+H	S	a	a	18.2% (16.3%, 20.3%)
	Dementia	38.6% (26.6%, 48.7%)	23.2% (17.0%, 29.9%)	18.3% (16.4%, 20.4%)
	Death	81.8% (74.8%, 86.8%)	62.1% (54.2%, 69.1%)	32.6% (30.2%, 35.1%)
D+S	H	a	a	33.0% (28.4%, 38.7%)
	Dementia	59.0% (0.0%, 83.6%)	31.8% (13.1%, 54.2%)	19.8% (15.9%, 24.7%)
	Death	86.4% (61.0%, 95.3%)	59.1% (34.5%, 77.1%)	19.5% (15.6%, 24.3%)
H+S	D	a	a	5.9% (4.8%, 7.4%)
	Dementia	52.4% (38.8%, 63.0%)	36.1% (27.5%, 44.8%)	20.9% (18.8%, 23.2%)
	Death	68.8% (60.3%, 77.0%)	46.2% (36.9%, 54.9%)	35.7% (33.2%, 38.3%)
D+H+S	Dementia	75.1% (40.0%, 89.7%)	51.8% (30.7%, 69.5%)	23.4% (20.1%, 27.3%)
	Death	77.8% (55.0%, 89.1%)	37.0% (18.8%, 55.3%)	41.5% (37.5%, 45.9%)

* For KMKaplan-Meier and CIFcumulative incidence function methods, the pattern of cardiometabolic conditions was determined up to the ages of 70–75 (ie, baseline ALSWHAustralian Longitudinal Study on Women’s Health survey). For multistate models, the progression of cardiometabolic conditions over the study period was captured. Cumulative incidence of outcomes as the next immediate condition was estimated.

a, not considered; D, diabetes; H, heart disease; S, stroke

The estimated cumulative incidence of dementia up to the age of 90 using the KM method declined when the competing risk of death was considered using the CIF method (table 3). For example, for women with the baseline pattern of ‘None’, the KM and CIF estimates of cumulative incidence of dementia were 35.7% (95% CI 34.6%, 36.8%) and 27.3% (26.4%, 28.2%), respectively (but these women may have developed cardiometabolic conditions during the study period). The Aalen-Johansen cumulative incidence of dementia as the first event for women with no cardiometabolic condition over the whole study period—not only at the baseline—was 11.0% (95% CI 10.4%, 11.7%).

Comparing the patterns representing one single cardiometabolic condition, the highest cumulative incidence was seen for women with the pattern of ‘S’. The KM and CIF estimates of cumulative incidence for those with ‘S’ at baseline were 56.6% (40.5%, 68.4%) and 31.7% (22.7%, 40.9%), respectively (but these women may have developed other cardiometabolic conditions before dementia). The Aalen-Johansen cumulative incidence of dementia as the second event for women who first had stroke (at baseline or some other time during the study period) was 17.9% (16.0%, 19.9%). Indeed, majority of women with the pattern of ‘S’ had a report of heart disease immediately after stroke (cumulative incidence 25.7%). Such changes in the pattern were ignored in KM and CIF methods.

The confidence intervals for the Aalen-Johansen estimates are much narrower than for KM or CIF estimates because they are based on data from more women; for example, only 101 women had ‘S’ at baseline (table 2) whereas 1420 women had ‘S’ as their only condition before a diagnosis of dementia (online supplemental figure S1).

### Estimates of HRs

No departure from the PH assumption was seen. The standard and time-varying Cox and Fine and Gray models show that, compared with women with the baseline pattern of ‘None’, women with the other seven patterns usually had a higher risk of incident dementia (table 4). Online supplemental table S4 shows how the choice of baseline changed the estimated HRs for dementia in the standard Cox regression and Fine and Gray models. Generally, HRs declined when the baseline patterns of cardiometabolic conditions were determined at older ages (online supplemental table S4).

**Table 4:** Risk of incident dementia for women with different patterns of cardiometabolic conditions

Pattern	Cox model	Time-varying Cox model	Fine and Gray model	Time-varying Fine and Gray model
None	1.00	1.00	1.00	1.00
D	1.32 (1.16, 1.50)	1.15 (1.02, 1.30)	1.05 (0.92, 1.20)	1.17 (1.04, 1.32)
H	1.21 (1.08, 1.37)	1.19 (1.10, 1.29)	1.04 (0.92, 1.18)	1.07 (1.00, 1.16)
S	1.64 (1.19, 2.26)	2.12 (1.89, 2.39)	1.13 (0.81, 1.58)	1.54 (1.35, 1.75)
D+H	1.13 (0.82, 1.54)	1.41 (1.26, 1.58)	0.69 (0.49, 0.97)	1.15 (1.02, 1.29)
D+S	2.38 (1.19, 4.78)	2.71 (2.17, 3.37)	1.02 (0.47, 2.20)	2.10 (1.65, 2.67)
H+S	1.49 (1.12, 2.01)	2.05 (1.83, 2.29)	1.20 (0.87, 1.66)	1.51 (1.34, 1.71)
D+H+S	2.64 (1.56, 4.47)	2.24 (1.91, 2.64)	1.93 (1.06, 3.52)	1.47 (1.23, 1.75)
BIC	75 588.3	75 079.9	79 945.3	79 618.4

BIC, Bayesian information criterionD, diabetes; H, heart disease; S, stroke

Compared with the standard or time-varying Cox models, the HRs estimated in the Fine and Gray models were lower. The BIC of the time-dependent models was lower than the models in which the pattern was treated as a fixed variable (ie, determined based on baseline reports) (table 4).

## Discussion

Despite the methodological advances in survival analysis, research on disease progression in multimorbidity is still dominated by the use of the KM method and Cox regression. In this research, we used progression from cardiometabolic multimorbidity toward dementia as an example to compare the performance of alternative modelling strategies. The practice of categorising the patients into different patterns from baseline reports of conditions or biological variables is common in other settings such as the progression from mental health disorders toward diabetes,^26^ from body mass index trajectories in childhood to cardiometabolic conditions in adulthood^27^,^29^ or from the trajectory of blood pressure toward cardiovascular diseases.^30^

Several studies have recommended adjustments for competing risks in fields such as cardiovascular disease, coronary diseases^9 31 32^ or nephrology,^16^ especially for elderly populations which may be more frail and at greater risk of competing events occurring before the main outcome.^33^ However, no study compared the performance of traditional methods with more advanced methods that take into account the progression of conditions over the follow-up time.

### Comparison of methods to estimate the cumulative incidence

Compared with CIF estimates, the estimated cumulative incidence in the KM method was biased upwards. This was consistent with findings from previous studies in other settings.^9 31 32^ In the KM and CIF methods, the only possible destinations for women with any of the baseline cardiometabolic patterns were dementia or death. This was not the case in the multistate model, as the method captures the progression of conditions after the baseline survey. For example, based on the multistate model, the majority of women with the pattern of ‘D’ (anytime during the follow-up) had a report of ‘H’ as their second immediate event (cumulative incidence=44.7%), indicating that women with the pattern of ‘D’ were likely to move to the pattern to ‘D+H’ before having a report of dementia (table 3).

An advantage of the Aalen-Johansen multistate method is to take into account the progression of conditions over the study period. This is important, especially for conditions with low prevalence at mid-age. For example, the number of women with the pattern of ‘S’ was 101 at baseline (contributed to KM and CIF analyses). However, 1420 women had ‘S’ as their only condition before a diagnosis of dementia (contributed to multistate analysis). Moreover, confidence intervals for the Aalen-Johansen estimates were narrower, compared with KM or CIF estimates (as the method captured the progression of conditions after baseline). This indicates that an advantage of the multistate model is that the method provides more accurate estimates as it incorporates information on reports of conditions over the whole study period into the analysis.

### Comparison of methods to estimate the HR

Compared with women with the pattern of ‘None’, women with other patterns were at higher risk of incident dementia. This was consistent with the results of previous studies (in which the baseline pattern was used as the exposure and the standard Cox regression as the modelling approach).^47 34^,^36^ Moreover, HRs in the Fine and Gray models (that consider the competing risk of death) were lower than in the Cox models. Only one of the five previous studies of the association between patterns of cardiometabolic conditions at baseline survey and risk of incident dementia compared results of the standard Cox model with the Fine and Gray model.^34^ Using the standard Cox model, relative to the participants with no cardiometabolic condition, the HRs for participants with one condition and with cardiometabolic multimorbidity (two or more conditions) were 1.42 (95% CI 1.27, 1.58) and 2.10 (1.73, 2.57), respectively. In the competing risk analysis, the HRs declined to 1.07 (0.97, 1.17) and 0.92 (0.77, 1.09).

In addition to the competing risk of death, Hu recently pointed out that multimorbidity tends to increase with age so that baseline patterns of conditions are likely to change over time.^8^ This issue is illustrated by comparing table 2 and online supplemental tables S2 and S3 which show how the distribution of cardiometabolic multimorbidity patterns among ALSWH participants changed from 1996 when they were aged 70–75 at the time of survey 1, to 2002 when they were aged 76–81 (corresponding to survey 3) and 2008 when they were aged 82–86 (corresponded to survey 5). Moreover, online supplemental table S4 showed a decline in the estimated HRs for dementia in the standard Cox or Fine and Gray regression when the baseline patterns of cardiometabolic conditions were determined at older ages (online supplemental table S4). This effect is also apparent in the literature.^37^ This same scenario of baseline patterns of the cardiometabolic conditions of diabetes, heart disease and stroke used to predict the subsequent risk of dementia has been examined by several authors.^47 34^,^36^ The HRs calculated by standard Cox models are shown in online supplemental table S5.

These examples show the importance of taking changing patterns of multimorbidity into account. This can be done in various ways. One approach is to use the different patterns of conditions as time-varying covariates or predictor variables in Cox or Fine and Gray models. However, there are alternative time-varying models with different assumptions. For example, each of the cardiometabolic conditions—but not the pattern—could be defined as a time-varying covariate. Alternatively, the conditions could be treated as recurrent events.^38^ To compare results from the time-varying and standard form of the models, for this paper the pattern of conditions was taken as a time-varying covariate. Our results found that the time-dependent models (that captured the progression of conditions over the study period) had better goodness of fit. The data layout required to apply advanced methods discussed in this paper is presented in online supplemental tables S6–S8.

Austin *et al* have urged caution in the interpretation of the estimates based of time-varying covariates, especially from a Fine and Gray model.^39^ They argued that such variables should be included if the value of the covariate is known for the entire time that the subject remains in the risk set. In our study, it was assumed that when a subject has a report of condition it remains switched on. This assumption is justifiable due to the chronic nature of the cardiometabolic conditions.

### Other methods

In this paper, we used baseline reports of cardiometabolic conditions and categorised the subjects into eight mutually exclusive patterns to address the pitfalls of traditional methods in which competing risks and the progression of conditions are not considered. Some other studies used group-based trajectory modelling or latent class growth analysis to categorise the participants according to the temporal patterns of longitudinal change of multimorbidity status based on the history of chronic conditions up to a baseline time.^3^ For example, Chen *et al* classified the subjects into mutually exclusive categories based on the speed of growth of conditions and estimated the risk of incident dementia using the standard Cox model. Methods based on grouping common trajectory patients have been used in other settings as well, for example, to examine the association between body mass index trajectories in childhood and the risk of cardiometabolic conditions in adulthood,^27^,^29^ or to study the trajectory of blood pressure and risk of cardiovascular diseases.^30^

The use of such methods in conjunction with the standard regression models results in the same shortcomings. This is because if the clustering or trajectory analysis had been performed at a different time point, the distribution of the trajectory categories could have changed. The aim of this paper was not to compare different algorithms to classify the subjects based on the history of conditions up to a time point, but rather to demonstrate the bias in estimation of risk when competing risks and progression of conditions are not considered.

Joint modelling of longitudinal and survival methods is an alternative method with a longitudinal component and a survival component.^40^ Usually, the longitudinal component examines the trajectory of a given continuous risk factor as a function of other risk factors using linear mixed regression models. For example, the method has been used to estimate the association between viral load and survival of HIV/AIDS patients.^41^ The authors examined the association between some risk factors and the trajectory of viral load (as a longitudinal outcome) using linear mixed regression, as well as the association between the same risk factors and the estimated trajectory of viral load on the survival of the patients (as the survival outcome). The application of such methods was beyond the scope of this study.

Another advanced method is latent transition analysis (LTA) in which several variables are used to extract latent classes and estimate latent transition probabilities from one latent class to another. Similar to the multistate models, the LTA relies on the Markov assumption.^42^ We did not apply this method as the patterns of cardiometabolic conditions were observable.

### Limitations

Our study had some limitations. First, differences in the periods when data were available for various conditions (online supplemental table S1) mean that the HRs and the cumulative incidence may have been underestimated. Second, the diagnosis of cardiometabolic conditions and dementia was based on self-report (from ALSWH surveys) or administrative health records. Therefore, some cases of cardiometabolic conditions or dementia may not have been diagnosed. For example, estimating the prevalence of dementia in 1921–1926 ALSWH women using the capture-recapture methodology found 2.7% underestimation in prevalence (20.4% in linked data vs 26.0% in the capture-recapture method).^43^ It should be noted that these limitations were consistent across all seven methods evaluated in this study. Therefore, the comparative analysis and the conclusions about the methods remain valid, as all methods were subject to the same constraints.

Another limitation of this study which may also be a strength was that the sample was only comprised of women. It has been shown that there are gender differences in terms of risk factors for cardiometabolic conditions^44^ and the patterns of cardiometabolic multimorbidity.^45^ Therefore, the estimates should not be generalised to the whole population.

In this research, left censoring (when an event occurs before the start of the study) and left truncation (when subjects at risk prior to baseline do not remain observable until the start of follow-up) may not be an issue because in the first ALSWH survey, women were asked ‘Have you ever been diagnosed with …?’. Therefore, events that had occurred before the ALSWH surveys were identified, and it was assumed that women were at risk from the starting date of the coverage period of the data sources used to extract records of conditions. It should be acknowledged that the ALSWH survey experienced moderate non-response rates during each follow-up. As summarised in online supplemental table S1, multiple sources of data were used to identify reports of conditions. Therefore, even if women were no longer completing the ALSWH survey, the incidence of hypertension, diabetes, stroke or dementia, could be identified using other sources.

### Conclusion

There are tutorials on the utility of competing risks and multistate models.^17 46^ Moreover, previous studies that applied multistate models mainly emphasised the risk factors that were associated with disease progression^47^,^49^ or cumulative incidence of being in each state.^50^ To our knowledge, this is the first study that has compared the performance of seven alternative methods to demonstrate the flexibility of multistate and time-varying models to investigate the progression from cardiometabolic conditions to dementia. In conclusion, the KM and Cox regression models do not consider the competing risks or the progression of exposure variables. On the other hand, multistate and competing risk models with time-varying covariates are flexible tools that make the most use of data allowing for the transitions between conditions over the study period. These models may provide further insight into the progression patterns of multiple chronic conditions.

## supplementary material

10.1136/bmjph-2024-000963 online supplemental file 1

## Data Availability

No data are available.
