# Supplementary material for: Comparison of seven models for the progression patterns of multiple chronic conditions in longitudinal studies
Source: BMJ Public Health. 2024 Nov 29;2(2):e000963. doi: 10.1136/bmjph-2024-000963 (PMC11816716; doi:10.1136/bmjph-2024-000963)
Supplement: online supplemental file 1 [file bmjph-2-2-s001.pdf]

Supplementary Table S1: List of conditions, criteria, and sources used (with coverage period) to identify patients

Source	Dementia	Diabetes	IHD	Stroke
Cause of Death<br>1996 to 2019	Reported once.	Reported once	Reported once	Reported once
MBS[1]<br>1984 to 2021		Reported one diabetes annual cycle of care items<br>**OR**<br>Three or more other specific diabetes procedures (Hb1Ac, eye exam for diabetes, allied health for diabetes).	Reported once or more.	
PBS[2]<br>2002 to 2021	Reported twice or more scripts within 12 months.	Reported two or more scripts within 12 months	Reported two or more scripts within 12 months	
Hospital admissions/ emergency[3], varied	Reported once or more.	Reported once or more.	Reported once or more.	Reported once or more.
Aged care[4], varied	Reported once or more.	Reported once or more.	Reported once or more.	Reported once or more.
ALSWH[5]<br>1996 to 2019	Reported once **BUT NOT** medication only.	Reported twice or more<br>**AND**<br>Once or more in MBS, PBS, Hospital, or Aged Care.	Reported twice or more<br>**AND**<br>Once or more in MBS, PBS, Hospital, or Aged Care.	Reported once or more<br>**AND**<br>Once or more in Hospital or Aged Care.

[1]MBS: Medicare Benefits Schedule

[2]PBS: Pharmaceutical Benefits Scheme

[3] Coverage of hospital data varied between States and Territories, starting between 1970 and 2007, and ending between 2017 and 2021.

[4] Coverage of aged care data varied between schemes, starting between 1997 and 2008 and ending between 2015 and 2020.

[5]The first ALSWH surveys used in this study were conducted in 1996. However, the text of the questions covered the whole life period.

In ALSWH surveys, for cardiometabolic conditions (i.e., diabetes, heart disease, and stroke), the question in survey 1 was "have you ever been told by a doctor that you have …?". From survey 2 onwards, the question was changed to "in the past three years, have you been diagnosed or treated for …?". The condition of Alzheimer's disease or dementia was asked from survey 2 onwards. These survey data were linked to administrative health records. These included hospital admissions; investigations procedures, medication prescriptions subsidised by the national health system scheme; assessments for aged care support; and medically certified causes of death. Hospital and emergency department data were obtained from each State and Territory, with some variation in coverage dates between jurisdictions. Data on investigations (e.g., glycosylated haemoglobin tests or angioplasty) were obtained from the universal health insurance scheme, Medicare (for items listed on the Medical Benefits Schedule, MBS). Similarly, data on government-subsidised medications were obtained from the Pharmaceutical Benefits Scheme (PBS) which is available to everyone. Additional data on diagnoses were available from various assessments for government supported aged care. Cause of death data including underlying and contributing causes obtained from multiple cause coded data.

Diabetes included type 1 and 2 diabetes mellitus and excluded gestational diabetes. Heart disease included heart surgery (heart bypass, angioplasty, and angiography) and acute coronary syndrome but not heart failure. Stroke included ischemic and haemorrhagic stroke. Dementia included Alzheimer's dementia, vascular dementia, and unspecified dementia.

Supplementary Table S2: Frequency (percentages) of women with each pattern of cardiometabolic condition based on the diagnosis of conditions up to 2002 (survey 3, ages 76 to 81)

	Number of women	Number of new cases of dementia	Number of deaths
None	6,845 (59.4%)	2,446 (35.7%)	5,373 (78.5%)
D	901 (7.8%)	331 (36.7%)	758 (84.1%)
H	2,252 (19.6%)	691 (30.7%)	1,994 (88.5%)
S	419 (3.6%)	138 (32.9%)	383 (91.4%)
D + H	617 (5.4%)	189 (30.6%)	591 (95.8%)
D + S	68 (0.6%)	23 (33.4%)	64 (94.1%)
H + S	295 (2.6%)	94 (31.9%)	272 (92.2%)
D + H + S	122 (1.1%)	31 (25.4%)	119 (97.5%)

Supplementary Table S3: Frequency (percentages) of women with each pattern of cardiometabolic condition based on the diagnosis of conditions up to 2008 (survey 5, ages 82 to 87)

	Number of women	Number of new cases of dementia	Number of deaths
None	4,421 (43.2%)	1,226 (27.7%)	3,263 (73.8%)
D	781 (7.6%)	238 (30.5%)	600 (76.8%)
H	2,608 (25.5%)	633 (24.3%)	2,218 (85.0%)
S	546 (5.3%)	132 (24.2%)	492 (90.1%)
D + H	877 (7.6%)	191 (21.8%)	793 (90.4%)
D + S	103 (1.0%)	30 (29.1%)	96 (93.2%)
H + S	617 (6.0%)	138 (22.4%)	571 (92.5%)
D + H + S	270 (2.6%)	59 (21.9%)	257 (95.2%)

Supplementary Table S4: Hazard ratio of incident dementia for ALSWH participants with different baseline patterns of cardiometabolic conditions

Diagnosis of cardiometabolic conditions up to:	Age at Survey 1 (70 to 75)	Age at Survey 3 (76 to 81)	Age at Survey 5 (82 to 87)
None	1.00	1.00	1.00
D	1.32 (1.16, 1.50)<br>*1.05 (0.92, 1.20)*	1.15 (1.03, 1.29)<br>*1.06 (0.94, 1.19)*	1.12 (0.98, 1.29)<br>*1.10 (0.96, 1.27)*
H	1.21 (1.08, 1.37)<br>*1.04 (0.92, 1.18)*	1.08 (0.99, 1.18)<br>*0.92 (0.84, 1.00)*	1.13 (1.03, 1.25)<br>*0.98 (0.89, 1.08)*
S	1.64 (1.19, 2.26)<br>*1.13 (0.81, 1.58)*	1.50 (1.27, 1.78)<br>*1.23 (1.03, 1.47)*	1.55 (1.29, 1.86)<br>*1.34 (1.12, 1.61)*
D + H	1.13 (0.82, 1.54)<br>*0.69 (0.49, 0.97)*	1.36 (1.17, 1.58)<br>*0.97 (0.82, 1.13)*	1.14 (0.98, 1.34)<br>*0.90 (0.77, 1.05)*
D + S	2.38 (1.19, 4.78)<br>*1.02 (0.47, 2.20)*	1.87 (1.24, 2.82)<br>*1.27 (0.83, 1.94)*	1.85 (1.29, 2.67)<br>*1.49 (1.03, 2.16)*
H + S	1.49 (1.12, 2.01)<br>*1.20 (0.87, 1.66)*	1.54 (1.25, 1.90)<br>*1.11 (0.89, 1.38)*	1.61 (1.34, 1.92)<br>*1.13 (0.94, 1.37)*
D + H + S	2.64 (1.56, 4.47)<br>*1.93 (1.06, 3.52)*	1.42 (1.00, 2.03)<br>*0.83 (0.56, 1.21)*	1.75 (1.35, 2.29)<br>*1.06 (0.80, 1.41)*

*: Pattern of cardiometabolic conditions was determined up to the ages at survey 1, survey 3, and survey 5.

**: The hazard ratios (HR) in the first and *second rows* corresponded to Cox regression and *Fine and Gray models*.

Supplementary Table S5: Summary of studies conducted on the association between different patterns of cardiometabolic conditions at baseline and risk of incident dementia

Reference	Wang et al. 2019		Dove et al. 2022		Tai et al. 2022		Dove et al. 2023		Chen et al. 2023	
Age:	73.6± 10.5		76.04± 10.0		64.9± 3.0		70.1± 7.5		64.1± 2.9	
Follow-up	Median 11 years<br>IQR: 5.7 to 11.7		Median 7 years		Median 12 years<br>IQR: 11.2 to 12.7		Median 15.4 years		Median 12.5 years<br>IQR: 11.6 to 13.2	
Event rate	291/ 2,648= 10.1%		155/ 704= 22.0%		4,766/ 203,038= 2.3%		3,020/ 17,913= 16.9%		5,750/ 204,646= 2.8%	
Pattern	N= 2,648		N= 704		N= 203,038		N= 17,913		N= 204,646	
None	1910<br>72.1%	1.00	398<br>56.5%	1.00	179,977<br>88.6%	1.00	13,762<br>76.8%	1.00	170,531<br>83.3%	1.00
Diabetes (D)	131<br>4.9%	1.02 (0.55, 1.90)	43<br>6.1%	0.42 (0.16, 1.07)	11,592<br>5.7%	2·03 (1·85, 2·23)	915<br>5.1%	1.51 (1.27, 1.79)	11,612<br>5.7%	1.71 (1.55, 1.88)
Heart disease (H)	349<br>13.2%	1.44 (1.05, 1.99)	149<br>21.2%	1.37 (0.91, 2.01)	5,881<br>2.9%	1·63 (1.43, 1·86)	1,846<br>10.3%	1.29 (1.12, 1.49)	13,709<br>6.7%	1.41 (1.29, 1.55)
Stroke (S)	96<br>3.6%	1.65 (1.03, 2.65)	23<br>3.3%	1.26 (0.59, 2.72)	3,163<br>1.6%	2·13 (1·82, 2·50)	551<br>3.1%	1.66 (1.34, 2.06)	3,733<br>1.8%	1.80 (1.55, 2.08)
D + H	85<br>3.2%	3.29 (2.01, 5.39)	51<br>7.2%	1.73 (0.97, 3.08)	1,341<br>0.7%	3·08 (2·50, 3·80)	355<br>1.9%	2.01 (1.49, 2.57)	3,155<br>1.5%	2.47 (2.15, 2.84)
D + S	10<br>0.4%	1.43 (0.18, 11.16)	4<br>0.5%	1.77 (0.80, 3.93)	526<br>0.3%	4·33 (3·28, 5·73)	99<br>0.5%	2.02 (1.10, 3.68)	550<br>0.3%	3.11 (2.34, 4.14)
H + S	50<br>1.9%	1.59 (0.81, 3.14)	27<br>3.8%	Not reported	420<br>0.2%	3·50 (2·50, 4·91)	310<br>1.7%	2.29 (1.68, 3.12)	1,000<br>0.5%	2.43 (1.94, 3.04)
D + H + S	17<br>0.6%	4.76 (2.04, 11.13)	9<br>1.3%	3.50 (1.23, 9.95)	138<br>0.06%	5·39 (3·30, 8·82)	75<br>0.4%	1.98 (0.99, 3.96)	356<br>0.2%	4.68 (3.40, 6.44)

Figure S1: Transition of women between cardiometabolic conditions before having a report of dementia

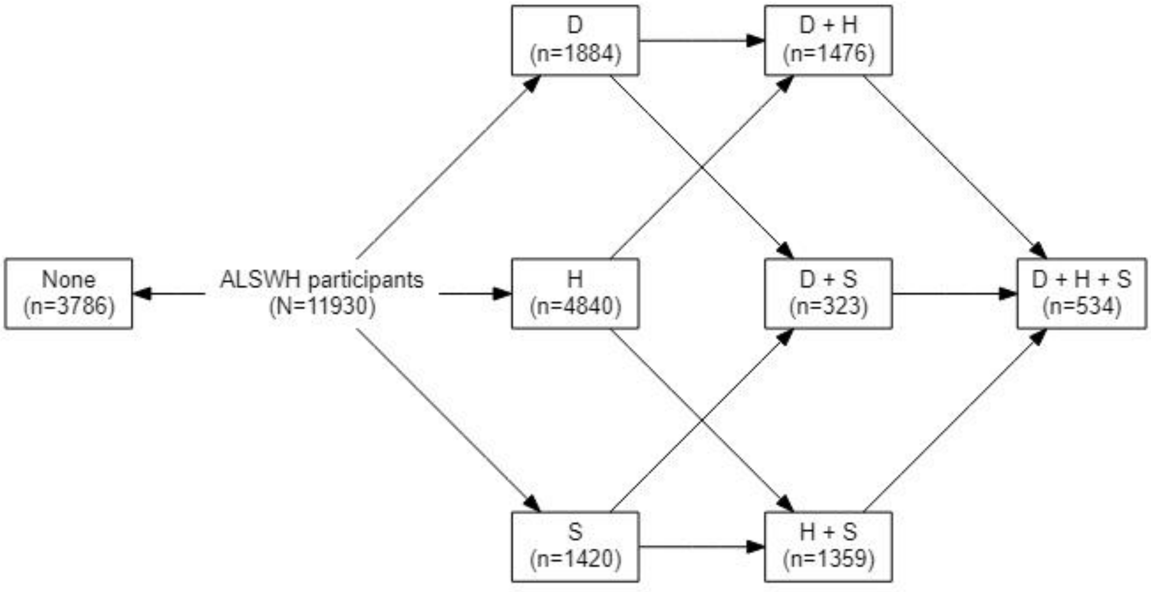

The number of women in each state is reported.

D: Diabetes (D); H: Heart disease (H); S: Stroke (S).

Data layout and sample codes

The standard dataset should have several columns for the diagnosis of each condition and age at diagnosis/ censoring. Table S6 provides data for three hypothetical women. There are five columns for diagnosis of diabetes, IHD, stroke, dementia, and death. These columns take values of 1 (for women with a report of each condition) and 0 (for censored women who had no report of the condition). The variable 'pattern_baseline' was calculated by comparing the age at diagnosis of three cardiometabolic conditions (i.e., diabetes, IHD, and stroke) and age at baseline survey.

ID 1: This case had records of diabetes and stroke. However, the age at the first report of both conditions was after her age at the baseline survey. Therefore, her baseline pattern was 'None'. She did not have a record of heart disease (ihd=0). The age at censoring for this event was defined as age at death.

ID 2: This case had a record of stroke at the age of 89.40. The age at the baseline survey was 68.5. Therefore, the baseline pattern was 'None'.

ID 3: This case had records of all three cardiometabolic conditions. Since the age at first report of stroke was after the age at baseline survey, the baseline pattern was defined as 'D + H' (which means diabetes and heart diseases).

Table S6: The standard data layout that can be used to fit models using baseline pattern

id	diabetes	age_diabetes	ihd	age_ihd	stroke	age_stroke	dementia	age_dementia	death	age_death	age_baseline	pattern_baseline
1	1	75.69	0	87.33	1	76.27	1	84.89	1	87.33	73.3	None
2	0	89.75	0	89.75	1	89.40	0	89.75	1	89.75	68.5	None
3	1	66.95	1	67.06	1	70.00	0	78.17	1	78.17	69.2	D + H

SAMPLE CODE TO FIT MODELS THAT USE BASELINE PATTERN:

**TRADITIONAL METHODS WHICH NEITHER CONSIDER THE COMPETING RISK OF DEATH NOR THE PROGRESSION OF CONDITIONS**
**Cox regression to estimate the hazard ratio (based on baseline pattern)**<br><br>library(survival)<br>hr_cox= coxph(Surv(age_dementia – age_baseline, dementia) ~ as.factor(pattern_baseline), data= mydata)<br>summary(hr_cox)
**KAPLAN-MEIER to estimate the cumulative incidence at selected ages (based on baseline pattern)**<br><br>library(survival)<br>incidence_km= survfit(Surv(age_dementia, dementia) ~ pattern_baseline, data= mydata)<br>summary(incidence_km, times= c(70, 75, 80, 85, 90))
**COMPETING RISK METHODS WHICH CONSIDER THE COMPETING RISK OF DEATH BUT NOT THE PROGRESSION OF CONDITIONS**
**Fine and Gray regression to estimate the hazard ratio (based on baseline pattern)**<br><br>library(survival)<br>library(riskRegression)<br>library(prodlim)<br><br>First, new variables for time and status should be created. The new status variable (named status_fg here), takes 1 for women with a report of dementia, 2 for women with no report of dementia who died, and 0 for other women.<br><br>mydata $status_fg=ifelse(mydata $dementia==1, 1,<br>ifelse(mydata $dementia==0 & mydata $death==1, 2, 0))<br><br>mydata $time_fg=ifelse(mydata $dementia==1, mydata $age_dementia, mydata $age_death)<br><br>There are alternative codes to fit the Fine and Gray subdistributional hazard model.<br>**FGR function:**<br>hr_fg <- FGR(Hist(time_fg - age_baseline, status_fg) ~ as.factor(pattern_baseline), data = mydata, cause=”dementia”)<br>hr_fg<br><br>**crr function:**<br>mydata$status_fg=factor(mydata$status_fg, levels=c(0,1,2), labels=c("censored","dementia","death"))<br><br>hr_crr <- crr(Surv(time_fg - age_baseline, status_fg) ~ as.factor(pattern_baseline), data = mydata)<br>print(hr_crr $tidy, n=100)<br>hr_crr

finegray function:

```
fdata1 <- finegray(Surv(time_fg - age_baseline, status_fg) ~ ., data = mydata, etype = 'dementia')

fgfit1 <- coxph(Surv(fgstart, fgstop, fgstatus) ~ as.factor(pattern_baseline), data = fdata1, weight = fgwt, x=TRUE, y=TRUE)
fgfit1
```

Cumulative incidence function to estimate the cumulative incidence (based on baseline pattern)

```
library(cmprsk)
library(tidycmprsk)

cum1=cuminc(Surv(time_fg, status_fg)~  pattern_baseline, mydata)
cum1
```

To fit the multistate model, the data should be expanded (Table S7).

SAMPLE CODE FOR MULTISTATE MODEL (JUST FOR THE FIRST TRANSITION)

At time 0, women were free of all conditions. There are five possible transitions (e.g., transition from birth to diabetes, .. or to death). The transMat function creates the matrix of possible transitions.

```
t_birth=transMat(x=list(c(2, 3, 4, 5, 6), c(), c(), c(), c(), c()),
             names=c("birth", "diabetes", "IHD", "Stroke", "dementia", "death"))
t_birth
```

```
 t_birth
             to
rom          birth diabetes IHD Stroke dementia death
  birth         NA        1   2      3        4     5
  diabetes      NA       NA  NA     NA       NA    NA
  IHD           NA       NA  NA     NA       NA    NA
  Stroke        NA       NA  NA     NA       NA    NA
  dementia      NA       NA  NA     NA       NA    NA
  death         NA       NA  NA     NA       NA    NA
```

The function msprep creates a matrix and counts the number of women with each transition. For example, for 1646 women the first reported condition was dementia. Moreover, 552 women did not report any of the five conditions.

```
ms_birth=msprep(data=m, trans=t_birth,
            time=c(NA,"age_diabetes","age_ihd", "age_stroke", "age_dementia", "age_death"),
            status=c(NA,"diabetes", "ihd", "stroke", "dementia", "death"),id="id",
            keep=c("diabetes", "ihd", "stroke", "dementia", "death",
                 "age_diabetes",     "age_ihd",     "age_stroke",     "age_dementia",     "age_death",
"age_baseline", " pattern_baseline "))
events(ms_birth)
```

```
             to
from          birth diabetes   IHD Stroke dementia death no event total entering
  birth           0     1884  4840   1420     1646  1588      552          11930
  diabetes        0        0     0      0        0     0     1884           1884
  IHD             0        0     0      0        0     0     4840           4840
  Stroke          0        0     0      0        0     0     1420           1420
  dementia        0        0     0      0        0     0     1646           1646
  death           0        0     0      0        0     0     1588           1588
```

```
covs=c("diabetes", "ihd", "stroke", "dementia", "death",
     "age_diabetes",   "age_ihd",   "age_stroke",   "age_dementia",   "age_death",   "age_baseline",
"baseline_baseline")
```

The expand.covs command expands the data in the format required for multistate modelling (Table S7). There were five possible transitions (shown in the trans column). The columns ‘from’ and ‘to’ shows the transition from the first state (i.e., when women were event free) to other states (codes 2 for diabetes, 3 for ihd, 4 for stroke, 5 for dementia, and 6 for death). The first destination for the woman with id= 1 was diabetes at the age of 75.69. Table S7 shows expanded data just for the first transition. Therefore, this woman was censored for other events at the age of 75.69. The column ‘status’ took 1 for the transition to diabetes and 0 for other transitions.

```
ms_birth=expand.covs(ms_birth, covs, longnames = FALSE)
```

The following codes can be used to estimate the cumulative incidence of transition from the state of event-free to each of the other states at selected ages:

```
c_birth <- coxph(Surv(Tstart, Tstop, status) ~ strata(trans), data = ms_birth, method = "breslow")
msf_birth <- msfit(object = c_birth, vartype = "greenwood", trans = t_birth)
pt_birth <- probtrans(msf_birth, predt = 0, method = "greenwood")
summary(object = pt_birth, times = c(80, 85, 90), conf.type = 'log')
```

Table S7: Data format required for multistate analysis (data shown only for the first transition)

id	from	to	trans	Tstart	Tstop	status	pattern_baseline
1	1	2	1	0	75.69	1	None
1	1	3	2	0	75.69	0	None
1	1	4	3	0	75.69	0	None
1	1	5	4	0	75.69	0	None
1	1	6	5	0	75.69	0	None
2	1	2	1	0	89.40	0	None
2	1	3	2	0	89.40	0	None
2	1	4	3	0	89.40	1	None
2	1	5	4	0	89.40	0	None
2	1	6	5	0	89.40	0	None
3	1	2	1	0	66.94	1	D + H
3	1	3	2	0	66.94	0	D + H
3	1	4	3	0	66.94	0	D + H
3	1	5	4	0	66.94	0	D + H
3	1	6	5	0	66.94	0	D + H

SAMPLE CODE TO FIT TIME-DEPENDENT COX AND TIME-DEPENDENT FINE AND GRAY MODELS
**Time-dependent Cox regression to estimate the hazard ratio (based on time-dependent pattern)**<br><br>Data should be prepared in a long format with two columns describing the time periods women experienced events (Table S8). For example, the woman with id= 1 experienced diabetes at the age of 75.69 and stroke at age of 84.89. Therefore, her data is presented in three rows. She did not have a record of dementia therefore the column status always took 0. The column status_fg is the same as the status. However, for women who died without a record of dementia, it took 2 instead of 0 (only in the last follow-up window). The column pattern shows the pattern of cardiometabolic conditions in each period window.<br><br>hr_cox_time_dep=coxph(Surv(Tstart, Tstop, status)~ as.factor(pattern), data=mydata_long)
**Time-dependent Fine and Gray regression to estimate the hazard ratio (based on time-dependent pattern)**<br><br>mydata_long $status_fg=factor(mydata_long $status_fg, levels=c(0, 1, 2), labels=c("censored", "dementia", "death"))<br>fdata1 <- finegray(Surv(Tstart, Tstop, status_fg) ~ ., i=id, data = mydata_long, etype ='dementia')<br>fgfit1 <- coxph(Surv(fgstart, fgstop, fgstatus) ~ as.factor(pattern), data = fdata1, weight = fgwt)<br>fgfit1

Table S8: Data layout for time-dependent Cox and time-dependent Fine and Gray models

id	Tstart	Tstop	status	status_fg	pattern
1	0	75.69	0	0	None
1	75.69	76.27	0	0	D
1	76.27	84.89	1	1	D + S
2	0	89.40	0	0	None
2	89.40	89.74	0	2	S
3	0	66.94	0	0	None
3	66.94	67.05	0	0	D
3	67.05	70.00	0	0	D + H
3	70.00	78.16	0	2	D + H + S

TECHNICAL NOTES

Material and Methods

Survival analysis is based on the time (shown by T) from a baseline until the occurrence of an event of interest. Two main functions in survival analysis are the hazard function (shown by h) and the survival function (shown by S).

Estimation of the cumulative incidence

In the absence of competing risks, the hazard function describes the instantaneous rate of occurrence of the event of interest in subjects who are still at risk of the event (Formula 1). The hazard function at the time t_j can be estimated by dividing the number of events by the population who is at risk (Formula 2).

$$Formula\ 1: h(t) = lim_{\Delta t \to 0} \frac{P(t \le T < t + \Delta t | T \ge t)}{\Delta t}$$

$$Formula\ 2: h(t_j) = \frac{d_j}{n_j}$$

The survival function at time t describes the probability of being event-free at least up to time t. The Kaplan-Meier (KM) method gives a simple estimate of the survival function (i.e., surviving up to time t_j) is the product of the probability of surviving up to time t_{j-1} and the conditional probability of surviving up to time t_j given being alive beyond t_{j-1} (Formula 3). In the absence of competing risks, one minus the KM survival function describes the cumulative incidence of the event.

$$Formula\ 3: S(t_j) = S(t_{j-1}) * \left(1 - h(t_j)\right) = S(t_{j-1}) * \left(1 - \frac{d_j}{n_j}\right) = \Pi_{t_j \le t}(1 - \frac{d_j}{n_j})$$

The KM method relies on the assumption of noninformative censoring. This means that individuals who are censored (i.e., are no longer contributing data after time t) are assumed to have the same future risk of the outcome. This assumption is violated when the reason for censoring is the occurrence of a competing event such as death. Therefore, KM estimates tend to be biased upward.

More generally, in the presence of competing risks, a person can experience one of K different events (including the outcome of interest). Assume status is an indicator variable denoting the type of event that occurred. In the presence of competing risks, the Fine and Gray subdistributional hazard function estimates the instantaneous risk of failure from the kth event in subjects who have not yet experienced an event of type k (Formula 4). The risk set includes subjects who are event-free as well as those who previously experienced a competing event.

$$Formula\ 4: h_k^{sd}(t) = lim_{\Delta t \to 0} \frac{P(t \le T < t + \Delta t,\ status = k | T \ge t \cup (T < t \cap K \ne k))}{\Delta t}$$

The cumulative incidence function CIF method allows for the estimation of the cumulative incidence while taking the competing risks into account. The cumulative incidence function (CIF) for the kth event (e.g., dementia) is an estimate of the probability of experiencing the kth event before time t and before the occurrence of any competing events (e.g., death), (Formula 5). Here $t_{(i)}$, $h_k(t_{(i)})$, and $S(t_{(i-1)})$ describe the i-th largest event time, cause-specific hazard function for event type k, and the KM estimate of the overall survival at the previous event time.

$$Formula\ 5\text{: } CIF_k(T) = P(T < t, \quad status = k) = \sum_{t_{(i)} \leq t} h_k(t_{(i)})\, S(t_{(i-1)})$$

In contrast to the competing risk models in which people can only experience one of the outcomes of interest or a competing event, the multistate model provides a flexible framework that captures the progression of events and allows transition between several events over the life course. The term state is used to describe any one or combination of events or conditions (e.g., corresponding to different multimorbidity patterns). Multistate models are described by two main quantities: transition hazard and transition probability. The hazard for the transition between one state and the next (say from state m to state n at time t) is the instantaneous rate of occurrence of the second state among people who are in the first state (Formula 6), [16]. Usually, Cox regression with separate baseline hazard functions for each transition is used to estimate the transition hazards for all possible transitions. To capture the effect of ageing and holding the Markov assumption, the survival time is calculated from the beginning of the study (called clock-forward) rather than the age at entry to the states (called clock reset). The estimated transition hazards are then used to obtain the Aalen-Johansen estimates of the cumulative incidence. This differs from the KM and CIF methods which estimate the cumulative incidence of the outcome from baseline pattern (i.e., ignoring changes in these patterns that may have occurred during the study period).

$$Formula\ 6\text{: } h_{mn}(t) = lim_{\Delta t \to 0} \frac{P((t \leq T < t + \Delta t | T \geq t)}{\Delta t}$$

Estimation of the hazard ratio

The Cox proportional hazards regression model relates the hazard function (i.e., rate of occurrence of the outcome, provided in Formula 1) to the independent variables (e.g., baseline patterns of multimorbidity).

Like the KM method, the Cox regression relies on the assumption of noninformative censoring. To overcome this limitation, competing risk models can be used such as that suggested by Fine and Gray. They defined a subdistributional hazard function to describe the instantaneous rate of occurrence of the kth event in people who have not yet experienced this event including those who have experienced a

competing event (provided in Formula 4). The estimated hazard ratios should be interpreted in terms of the relative incidence of the outcome rather than hazards.

Both Cox and Fine and Gray models can be used to estimate relative hazards of the outcome of people with various combinations of conditions at baseline compared to the rate for people with none of these conditions. However, these analyses ignore the progression of multimorbidity during the study period. This limitation can be overcome using the time-varying Cox regression and time-varying Fine and Gray models. For these models, the patterns of conditions are treated as time-varying covariates.
